# Parents' and child health professionals' attitudes to dietary interventions in autism spectrum disorder (ASD): findings from a UK survey

**DOI:** 10.1186/1745-6215-14-S1-P23

**Published:** 2013-11-29

**Authors:** Elaine McColl, Ann Le Couteur, Sandra Adams, Helen McConachie, Paul Gringras, Gillian Baird, Anne O'Hare, David Wilson, Jeremy Parr, Elizabeth Winburn, Jenna Charlton

**Affiliations:** 1 Newcastle University, Newcastle upon Tyne, UK; 2 Northumbria Healthcare NHS Foundation Trust, North Shields, UK; 3 Guy's and Thomas' NHS Foundation Trust, London, UK; 4 Edinburgh University, Edinburgh, UK

## Background

Parents of children with ASD are increasingly using special diets and dietary supplements, despite a lack of robust evidence of effectiveness. The most popular dietary intervention is the gluten free casein free (GFCF) diet which is not without risks for the child and family.

## Objectives

To explore the attitudes of parents and health professionals towards dietary interventions and the use of the GFCF diet. To assess the feasibility of an RCT of this diet in preschool children with ASD.

## Methods

Short web-based questionnaire for parents and child health professionals.

## Results

246/361 parents and 246/317 professionals responded. Of all parent respondents, 46% were currently using dietary supplements for their child, 84% were aware of the GFCF diet and 28% were currently using this diet. Three quarters of parent respondents said they would ‘definitely', or would ‘consider' participating in an RCT of the GFCF diet.

72% of child health professionals had been approached by parents for advice about the GFCF diet. 50% of professionals reported they did not know enough about the efficacy of the diet to advise families. The majority of professionals strongly supported the need for evaluation of the GFCF diet and 75% would be prepared to recruit children to an RCT.

## Conclusions

The need to evaluate the GFCF diet has been confirmed. There is support amongst professionals and parents for an RCT of this diet and facilitators and barriers of recruitment and retention of families in a future RCT have been identified.

## Funding

Autism Speaks US.

